# Implementation feasibility of animal-assisted therapy in a pediatric intensive care unit: effectiveness on reduction of pain, fear, and anxiety

**DOI:** 10.1007/s00431-023-05284-7

**Published:** 2023-11-08

**Authors:** Eduardo López-Fernández, Alba Palacios-Cuesta, Alicia Rodríguez-Martínez, Marta Olmedilla-Jodar, Rocío Fernández-Andrade, Raquel Mediavilla-Fernández, Juan Ignacio Sánchez-Díaz, Nuria Máximo-Bocanegra

**Affiliations:** 1 Pediatric Intensive Care Unit. Hospital 12 de octubre, Madrid, Spain; 2 Pediatric Intensive Care Unit, Great Ormond Street Hospital for Children, London, UK; 3 PsicoAnimal, Madrid, Spain; 4 Animals and Society Chair Program, Faculty of Health Sciences, Rey Juan Carlos University, Madrid, Spain

**Keywords:** Animal-assisted therapy, Intensive care, Humanization, Pain, Fear, Anxiety

## Abstract

Animal-assisted therapies are an innovative strategy within health care humanization initiatives, and they could play a role in the reduction of pain or anxiety. The main objective of this work was to evaluate the feasibility of implementing animal-assisted therapy in a pediatric intensive care unit and its effectiveness for the reduction of pain, fear, and anxiety. A prospective, quasi-experimental study of animal-assisted therapy was designed in the pediatric intensive care unit of the Hospital Universitario 12 de Octubre of Madrid, from January 2019 to December 2019. The study sample included patients who had been admitted to the unit and were over 3 years old. Satisfaction surveys were collected from the patients, family, and health personnel involved. Physiological variables and the level of pain (visual analog scale or Wong-Baker scale), fear (Child Medical Fear Scale), and anxiety (modified Yale Preoperatory Anxiety Scale) were evaluated before and after each session. Any existence of adverse events was recorded. A total of 74 therapy sessions were performed on 61 patients. All sessions were completed without any adverse effects. A total of 164 surveys were collected, providing an overall project rating of 9.69 out of a possible 10. The survey comments were found to be positive in most cases. No differences were found in the physiological variables measured before and after each session. There was a statistically significant decrease in pain, fear, and anxiety levels (*p* < 0.01).

*  Conclusion*: The implementation of an animal-assisted therapy project in a pediatric intensive care unit is feasible and safe and has a high degree of acceptance among both participants and healthcare staff. Animal-assisted therapy is effective for the reduction of pain, fear, and anxiety, and therefore, it could be considered an adjunct to non-pharmacological therapy.

**Table:** 

**What is Known:** *• Animal assisted therapies (AAT) are an innovative strategy that could be beneficial to help pediatrics patients cope with admission difficulties and could even play a role in reducing pain, anxiety and/or delirium.* *• To date there are not studies to analyze the effectiveness of AAT in the field of Pediatric Intesive Care.*	
**What is New:** *• Our study confirms the feasibility and effectiveness of the implementation of an AAT in the field of Pediatric Intensive Care with a high degree of acceptance by participants, caregivers and healthcare personnel.* *• AAT demonstrated a reduction in pain, fear and anxiety in pediatrics patients admitted to Pediatric Intensive Care Unit.*

**What is Known:**

*• Animal assisted therapies (AAT) are an innovative strategy that could be beneficial to help pediatrics patients cope with admission difficulties and could even play a role in reducing pain, anxiety and/or delirium.*

*• To date there are not studies to analyze the effectiveness of AAT in the field of Pediatric Intesive Care.*

**What is New:**

*• Our study confirms the feasibility and effectiveness of the implementation of an AAT in the field of Pediatric Intensive Care with a high degree of acceptance by participants, caregivers and healthcare personnel.*

*• AAT demonstrated a reduction in pain, fear and anxiety in pediatrics patients admitted to Pediatric Intensive Care Unit.*

## Introduction

Relationships between humans and animals, especially dogs, have been documented for thousands of years. The first evidence of canine domestication dates from around 12,000 years ago [1]. Several articles to date have examined the interactions between hospitalized pediatric patients and animals, as well as their use to combat stress, pain, loneliness, or chronic diseases [2–8].

Animal-assisted interventions (AAI) are defined as those strategies in which an animal is introduced and becomes an active part of the activity in order to achieve different objectives (improvement of mobility, acquisition of social skills, reduction of pain or anxiety, etc.).

AAI can be divided into animal-assisted activities (AAA) whose objective is to improve the quality of life of the participants using an animal companion, or animal-assisted therapies (AAT) that represent a branch of therapeutics for different pathologies. AAT is defined as a “type of treatment, where the animal is an integral part of the process” [9].

This type of therapy is determined by a professional who sets specific treatment goals; furthermore, the whole process must be supervised and evaluated by the professional. Therefore, the main objective is to make the patient feel more at ease, and thus achieve the therapeutic objectives.

Advances in the field of intensive care medicine have led to higher survival rates in patients, but also an increase in associated morbidity. Moreover, in recent years, the characterization of Pediatric Post-Intensive Care Syndrome (p-PICS) [10] has emerged, defined as a series of changes across different areas (physical, cognitive, mental) that occur after admission to pediatric intensive care units (PICU).

Admission into the PICU can be a difficult situation for both patients and their families [11, 12]; therefore, it is essential to achieve PICU with a high degree of “humanized care,” based on the presence of family members with an open door policy, active involvement in patient care, the use of effective communication with the implementation of family-centered passes, or the presence of respite spaces for family members [13, 14]. In recent years, the study of alternative non-pharmacological therapies such as music therapy in neonatal units has also been expanded [15], or the implementation of early rehabilitation projects [16] and the use of ICU journals [17, 18] for the prevention and treatment of p-PICS.

The use of AAT could be beneficial to help patients cope with admission difficulties and could even play a role in reducing pain, anxiety, and/or delirium [19]. The implementation of an AAT program in a pediatric ICU requires its integration into a broader scheme involving the measures mentioned in the previous paragraph. It is necessary to have a referent in charge of coordinating the health professionals with the professionals in charge of the therapy and the patients. Likewise, it is essential to control the possible risks derived from the therapy, using certified professionals and animals that meet all the relevant health and safety conditions, limiting the number and duration of the sessions, and carrying out an adequate inclusion of patients, excluding those with a high risk of serious infections (immunosuppressed) or those colonized by resistant microorganisms [20].

To date, there are few studies on the use of AAI in intensive care units, and none of these studies has analyzed the feasibility and effectiveness of AAT in the field of pediatric intensive care [21]. In addition, this type of work could provide new evidence on how the human-animal bond can help in situations of vulnerability in childhood to improve their care and hospital attention.

## Aims

The principal aim of this study was to determine the feasibility of the implementation of an AAT program in PICU at the Hospital Universitario 12 de Octubre, Spain.

The secondary aim was to describe the satisfaction levels of all those involved in the sessions, and to analyze the changes in vital signs, pain, fear, and anxiety scales, which were measured before and after each intervention.

## Materials and methods

### Study design and period

A prospective, quasi-experimental, non-randomized study of AAT was designed and undertaken at the PICU of the Hospital Universitario 12 de Octubre, in Madrid, Spain, from January 2019 to December 2019.

The SQUIRE (Standard for Quality Improvement Reporting Excellence) guidelines were followed for the preparation of this paper.

### Setting

Hospital Universitario 12 de Octubre has a third-level PICU. It has 16 boxes, and attends some 400–500 patients per year, including patients with critical medical pathology of different etiology, liver transplantation, and congenital heart disease in pre- and postoperative stages, and patients with atherosclerosis. This unit treats patients from the neonatal period up to 17 years of age.

### Data collection procedure

The study was conducted in collaboration with the Chair of “Animals and Society” of the Universidad Rey Juan Carlos and the “*PsicoAnimal*” Association. The Research Chair was founded in 2016 at the Universidad Rey Juan Carlos. The objectives of this Research Chair include the study of the human-animal bond, how it is developed, and the benefits for all participants. For the development of research projects, this Chair collaborates with professional associations that provide the human-animal team for the implementation of the therapeutic sessions. For this project, collaborations were made with the “*PsicoAnimal*” Association. Dog-assisted therapy was carried out on a weekly basis and two 45-min sessions were held every day. Between both sessions, there was a break according to the hygiene and rest protocol of the human-animal therapeutic team. In most cases, the session was individual and performed in the patient’s own box, although there were sessions in which a group of patients was included (never including more than three people). In this case, the session was held in a room attached to the unit. The intervention group consisted of an occupational therapist (OT), a psychologist (PS), and a therapy dog. Both the OT and PS were also AAI technicians. Before each session, a meeting was held together with the healthcare team, where the decision was made relating to which patients/intervention candidates would be selected for that particular day, in addition to the therapeutic objective for each session.

A Golden Retriever (Zenit) and a Deutsch-Drahthaar (Senna) were used as therapy dogs for AAT sessions. The two animals underwent periodic check-ups by a veterinarian and met all relevant health criteria: vaccinations, grooming, treatment for parasites, and screening for enteric pathogens.

All interventions were directed by AAI technicians who chose the exercises and which animal would be used, taking into consideration the different characteristics of both dogs.

Zenit and Senna had different personality traits. Zenit was a calmer dog with many skills related to what is known as a “blanket dog” activity (an exercise in which the dog lies close to the patient to offer comfort and relaxation), whereas Senna’s personality was more active. After the intervention, the cubicle was cleaned, and the bed linen changed. The information collected included demographic data regarding the patient’s history, current illness, location of the session, presence of the caregiver during the intervention, and presence of any pets at home.

### Sample

The inclusion criteria were patients who were admitted to the PICU, aged between 3 and 17 years old, and who were able to actively interact in therapy, according to the established exclusion criteria.

In the event that there were no candidates present in the PICU who met these criteria, patients who had been admitted to other hospital services were included, giving preference to those who had previously been admitted to the PICU or those undergoing prolonged hospitalization.

The exclusion criteria were as follows: allergy or fear of dogs, neutropenia (defined by a concentration of neutrophils less than 500 cells/mL), severe immunodeficiency, moderate or deep sedation that made interaction with the animal impossible, aggressive behavior, or lack of consent.

A sample size calculation was not performed since the limiting aspect was the budget allocated to the project, which meant that only two to three weekly sessions could be carried out for a duration of 1 year. Therefore, we tried to recruit as many patients as possible under these circumstances; however, although it would have been desirable, it was impossible to increase the sample size.

### Variables, instruments, and data collection

The information collected included demographic data regarding the patient’s history, current illness, location of the session, presence of the caregiver during the intervention, and presence of any pets at home. Physiological variables (heart rate (HR), respiratory rate (RR), blood pressure (BP), oxygen saturation (OS)), as well as pain (Wong-Baker (WB), or visual analog scale (VAS)), fear (Child Medical Fear Scale (CMFS)), and anxiety scales (Modified Yale Preoperative Anxiety Scale (m-YPAS)), were collected before and after the intervention. The data collection was performed by a physician responsible for the unit, previously trained in the use of these scales, which were administered before the patient saw the animal and just after the end of the session. Concerning the physiological variables, the patients were under continuous monitoring of HR, RR and satO_2_, and therefore, these variables were collected before and after each session.

The Wong-Baker scale is a subjective scale that represents drawings with faces of different pain intensities (from 0 to 10 points); it is used in patients from 3 to 7 years old and the patient indicates with which face he/she feels more identified. The VAS consists of a graduated horizontal line of 10 cm, in which the extremes represent the extreme expressions of pain (on the left, the absence of pain; and on the right, the pain of greater intensity); the patient points to the point of pain and a measurement is made from 1 to 10 cm [22, 23]. The CMFS is a scale adapted from the Faces Anxiety Scale [24] for the measurement of fear in children undergoing painful medical procedures; it is also a subjective scale with different faces in which the child feels identified (scored from 0 to 4 points) [25]. The m-YPAS gathers the patient’s assessment in relation to five domains (activities, vocalization, emotional expressivity, apparent alertness, and interaction with family members) [26], before receiving a surgery. Given that this scale has been validated in Spanish [27] and it can be used for both preschool and school children (2–12 years), it was considered an acceptable scale for intensive care units, where painful procedures are performed, similar to those that could be performed as pre-surgical conditioning (e.g., peripheral cannulation, bladder catheterization, nasogastric tube insertion).

A subjective satisfaction survey was designed ad hoc by a committee of experts according to previous literature [6]. This committee was formed by an intensivist, a clinical health psychologist, and an expert in human-animal bonding. The survey was delivered to patients, caregivers, AAI therapy technicians, and other healthcare personnel participating in the session, meaning that more than one survey was collected for each intervention.

For the evaluation of feasibility, the staff satisfaction surveys were taken into account, as well as the possible adverse effects (extravasation via extravasation, mobilization of drains or probes, falls, desaturation, delays in medication administration) that could occur during therapy.

### Statistical analysis

Three consecutive analyses were performed in order to reduce possible confusion biases caused by the presence of interventions performed in other hospital services or of those two patients who received continuous intravenous analgesia. Thus, an overall analysis was performed, as well as an analysis by groups (those who received therapy in the ICU and those who received it outside the unit) and, finally, an analysis excluding those two patients who received continuous intravenous analgesia. A Kolmogorov–Smirnov or Shapiro–Wilk test for normality of data was used depending on the number of observations. The results obtained were expressed as a mean average and typical deviation for continuous normal variables, and as a median and interquartile range (IQR) for continuous non-normal variables. Hypothesis testing for pre- and post-intervention results was carried out using a Student *t*-test for paired samples in the case of normal variables, in addition to using the Wilcoxon test or the sign test for non-normal variables. Categorical variables were expressed as counts or percentages. Values with a  p < 0,05 were considered statistically significant. All statistical analyses were performed using the SPSS v program 22.

### Ethical considerations

The study was approved by the hospital’s Ethics Committee. Furthermore, consent was obtained from the patients’ parents prior to the beginning of the sessions.

In the case of patients over 12 years old, their informed consent was also obtained, whereas in patients between 6 and 12 years old, their informed assent was obtained.

The entire consent process and research procedures were carried out in accordance with Spanish legislation.

## Results

During the study period, a total of 74 interventions were performed with 61 patients. The median age was 8.6 years [IQR: 4.8–15.6], the majority of which were male (57%, 35). The main cause of admission was oncological disorders (34%, 21), followed by patients undergoing cardiac surgery (26%, 16). Most of the interventions were performed in a PICU cubicle (59%, 44), whereas in the case of patients who were able to stand independently, they were carried out in an adjoining room at the unit (24%, 17). Thirteen interventions were performed at other hospital services including patients who had been previously admitted to the PICU (Table 1).

**Table 1:** Sociodemographics and clinical variables

	**Median; interquartile range/frequency**
**Sociodemographics variables**	
** Age (years)**	8.6 [IQR 4.8–15.6]
**Male (%, *n*)**	57%, 35
**Cause of admission (%, *n*)**	
**- Oncological**	34%, 21
**- Cardiac surgery**	27%, 16
**- Infectious**	5%, 3
**- Neurological**	3%, 2
**- Others (include respiratory)**	31%, 19
**Site of the intervention (%, *n*)**	
**- PICU cubicle**	59%, 44
**- Adjoining PICU room**	24%, 17
-** Other**	17%, 13
**Owned domestic PET (%, *n*)**	46%, 28
**Duration of intervention (min)**	38 [35–45]
**Continuous infusion during intervention (%, *n*)**	59%, 44
**- Serotherapy**	50%, 37
**- Analgesia**	3%, 2
**- Vasoactive drugs**	1%, 1
**- Chemotherapy**	1%, 1
**- Others**	4%, 3
**Physiological variables (mean; standard deviation)**	**Pre-intervention**
**Heart rate (bpm)**	108 ± 18.8
**Respiratory rate**	27 ± 6.2
**Oxygen saturation (%)**	98 ± 1
**Median arterial pressure (mmHg)**	75 ± 12.8
**Behavioral variables (median; interquartile range)**	
**Wong-Baker**	2 (0–4)
**VAS**	4 (0–6)
**CMFS**	1 (0–2)
**M-YPAS**	38 (31–46)

*BPM *beats per minute, *CMFS *Children Medical Fear Scale, *m-YPAS *modified Yale Preoperatory Anxiety Scale *VAS *visual analog scale

Almost half of the participants (46%, 28) owned a domestic pet; however, none of them had had previous experience with AAT. The median duration of the interventions was 38 min [IQR: 35–45]. During the interventions, over half of the participants (60%, 44) were receiving continuous intravenous infusions; however, only two patients were receiving accompanying intravenous analgesia.

There were no statistically significant differences in terms of changes in physiological variables (heart rate, respiratory rate, blood pressure, oxygen saturation) before and after the intervention (see Table 2).

**Table 2:** Physiological and behavioral variables of participants pre- and post-intervention

	**Pre-intervention**	**Post-intervention**	***p* value**
**Physiological variables (mean; standard deviation)**			
** Heart rate (bpm)**	108 ± 18.8)	107 ± 14.5	0.453
** Respiratory rate**	27 ± 6.2	26 ± 5.1	0.946
** Oxygen saturation (%)**	98 ± 1	98 ± 2	0.251
** Median arterial pressure (mmHg)**	75 ± 12.8	74 ± 5.4	0.370
**Behavioral variables (median; interquartile range)**			
** Wong-Baker**	2 (0–4)	0 (0–2)	< 0.001
** VAS**	4 (0–6)	0 (0–2)	< 0.001
** CMFS**	1 (0–2)	0	< 0.001
** M-YPAS**	38 (31–46)	23 (23–25)	< 0.001

*BPM *beats per minute, *CMFS *Children Medical Fear Scale, *m-YPAS *modified Yale Preoperatory Anxiety Scale, *VAS *visual analog scale

In the analysis of the overall sample, the median pain score before the intervention was 2 points [IQR 0–4] on the WB scale and 4 points [IQR 0–6] on the VAS. After the intervention, a statistically significant decrease was evident, with a score of 0 points [IQR 0–2, *p* < 0.01] on the WB scale and 0 points [IQR 0–2, *p* < 0.01] on the VAS.

A statistically significant reduction was also evident in terms of the decrease in the level of fear on the CMFS scale from 1 point [IQR 0–2] prior to the intervention to 0 points (*p* < 0.01) after the intervention. The median anxiety score on the m-YPAS scale, prior to the intervention, was 38 points [IQR 31–46] and 23 points [IQR 23–25] after the intervention; this difference was statistically significant (*p* < 0.01).

When the two patients who were receiving continuous sedoanalgesia were removed from the analysis, statistical significance was maintained regarding reductions in the pain (WB and VAS), fear, and anxiety scales.

In the analysis by subgroups, those patients who were treated in a place other than the ICU box had a statistically significant decrease in pain measured using the VAS scale from an initial score of 3.5 [IQR 0–4.75] to 0 points [IQR 0–2] (*p* = 0.41). They also presented a statistically significant decrease in the level of anxiety on the m-YPAS scale from 32 points [IQR 31–38] before the intervention to 23 points [IQR 23–24] after the intervention (*p* = 0.027). Regarding fear assessment, a statistically significant difference is found in the reduction of the CMFS scale from 1 point [1, 2] to 0 points (*p* = 0.01).

In the subgroup performing therapy in the PICU box, the decrease in scores on the pain, anxiety, and fear scales was also maintained (Table 3).

**Table 3:** Comparison of behavioral variables between PICU group vs. extra-ICU group

**Median; interquartile range**	Pre-intervention	Post-intervention	*p* value
**Box PICU group**			
** Wong-Baker**	2 (0–4)	0 (0–2)	< 0.001
** VAS**	5 (1.5–6)	0 (0–2)	0.001
** CMFS**	1 (0–2)	0	< 0.001
** m-YPAS**	40 (33–46)	23 (23–31)	< 0.001
**Extra PICU group**			
** VAS**	3.5 (0–4.75)	0 (0–2)	0.41
** CMFS**	1 (1–2)	0	0.01
** m-YPAS**	32 (31–38)	23 (23–24)	0.027

*BPM *beats per minute, *CMFS *Children Medical Fear Scale, *m-YPAS *modified Yale Preoperatory Anxiety Scale, *VAS *visual analog scale

All procedures were completed and there was no evidence of any adverse effects of any relevance.

A total of 167 surveys were collected among the participants (13%, 23), caregivers (28%, 47), animal therapy technicians (40%, 67), and healthcare personnel (18%, 30). The overall satisfaction of the project was rated at 9.69/10, with a satisfaction rating among the participants of 9.53/10 and 9.38/10 among the caregivers (see Table 4). Most of the respondents felt that the possibility of receiving ATT could be a determinant factor influencing the choice of hospital (3.63/4) and they agreed that they would consider ATT to other patients in the future (3.93/4). The health staff did not perceive the activity to be an extra burden to their daily routine (1.41/4). Virtually none of the respondents believed that the intervention could be harmful to patients (1.25/4); however, the greatest concern was regarding the possibility of increased infection rates.

**Table 4:** Satisfaction questionnaire

	**Scoring: totally agree (4 points); agree (3 points); disagree (2 points); totally disagree (1 point)**			
	**Participant**	**Caregivers**	**Health care professional**	**Animal therapist**
**I am satisfied with the program**	3.69	3.95	3.92	4
**I think it is important for children**	3.88	3.92	3.88	4
**It fulfilled my expectations**	3.81	3.79	3.85	3.95
**I would recommend it to other patients**	3.94	3.82	3.88	4
**It could influence my choice of hospital**	3.44	3.05	3.62	4
**The program is well structured**	3.81	3.77	3.73	3.95
**There could be negative consequences for the patients**	1.31	1.51	1.38	1

**Only for staff**				
**It has a positive influence in daily work**	3.90			
**It is harmful for unit work**	1.21			
**It increases workload in the daily routine**	1.41			

	**Participant**	**Caregivers**	**Health care professional**	**Animal therapist**
**Number**	17	39	28	63
**Global satisfaction (over 10 points)**	9.53	9.38	9.50	10

Overall, the comments in the open questions section of the survey were mostly positive. These comments mainly highlighted the achievement of greater patient comfort, as well as the opportunity for distraction and evasion from the intensive care environment, the generation of spontaneous smiles, and increased social interaction. Examples of these comments include the following: “Time goes by faster. There is a motivation. It’s a great experience. It serves as an emotional release. The interaction with the dog and therapists is very enriching. It serves as a learning experience” (questionary 36); “Very beneficial for the patients: distraction, calm and comfort. To me, it seems a perfect non-pharmacological treatment for anxiety. Well-being among the health personnel accompanying and assisting the therapy” (questionary 29); “Improvement of the comfort and general condition of the child. No previous interaction with staff and since the intervention he has smiled for the first time since his admission and has been more confident with us. From complaining of pain prior to the intervention, to no pain after” (questionary 17). The negative comments were mainly based on the excessive presence of staff during the interventions and the short duration of the activity.

## Discussion

Our study demonstrated a decrease in the level of pain pre- and post-intervention. Although the pain level prior to the intervention was low (2 points on the WB scale and 4 points on the VAS), the reduction in pain was still statistically significant. These findings are consistent within the current body of literature where other studies have shown benefits in terms of pain reduction when using AAI in pediatric patients [3, 4, 28, 29]; however, none of these studies had been previously undertaken in a PICU. Braun et al. [3] found a reduction in pain levels of 2.86 points, with no differences in physiological variables except for an increase in respiratory frequency. Calcaterra et al. analyzed the use of AAI in the postoperative setting, finding a lower perception of pain in the intervention group, although there were no changes in the administration of analgesia. In the systemic review and meta-analysis conducted by Feng et al. [29] including a total of eight studies (four randomized clinical trials and four quasi-experimental) with a total of 348 hospitalized pediatric patients, a statistically significant decrease in pain levels (− 0.49; 95% confidence interval [CI] 0.77 to − 0.22; *p* < 0.001) was reported, with no differences in anxiety, depression, or stress levels.

Anxiety and fear are other important factors that can affect children during their admission to the PICU. These variables are difficult to estimate; however, scales such as the CMFS and the m-YPAS have been developed in order to quantify them. Our participants presented a statistically significant score reduction in both scales, which is an interesting discovery compared to previous studies [5, 28–30]. Tsai et al. [5] included 15 hospitalized pediatric patients in whom they performed an AAT intervention without finding significant differences in anxiety scores. Branson et al. [30] conducted a randomized clinical trial involving 48 hospitalized pediatric patients (24 in each group) to whom an AAA intervention is applied, reporting a trend toward decreased anxiety but unable to prove it statistically. On the other hand, Barker et al. [28] designed a randomized controlled trial that included a total of 40 hospitalized pediatric patients, one group receiving AAI and the other receiving active control condition, showing a decrease in anxiety levels in both groups pre- and post-intervention, but without finding differences between the groups. Finally, the meta-analysis by Feng et al. [29] which includes the previous studies, although it shows a trend toward a decrease in anxiety, does not demonstrate this in a statistically significant manner. Although the samples of the different studies are heterogeneous, our results may provide further information in a new setting such as pediatric intensive care.

A questionnaire was developed to obtain an objective evaluation of the satisfaction of those involved. A high degree of satisfaction was found in all categories of the survey. The overall satisfaction of participants (3.69/4) and that of their family members (3.95/4) are consistent with those of previous studies [6, 31–33] in which a satisfaction rate of over 90% was observed, with a near 100% rate of recommendation to other patients. Regarding the comments in the open questions section of the survey, the participants and their caregivers reported that they found it to be a very entertaining way to overcome the anxiety caused by admission to the PICU, and a good way to create a more similar environment to that found in their natural environment. Consequently, the experience was perceived as excellent by most of the participants. In relation to the healthcare personnel, overall satisfaction was extremely high (9.50/10). ATT was implemented as an additional activity in the daily routine and was therefore not perceived as an increased burden to their workload. Teamwork is a fundamental element in the PICU and this was one of the keys to the success in the project.

All the planned interventions were concluded without complications. The biggest concern of both the participants and the healthcare personnel was the possibility of an increase in infection rates following the activity, despite the fact that previous studies have shown that this is not the case [6, 7, 34, 35]. Using data from the ENVIN (National Nosocomial Infection Surveillance Study), as a reference, no increase in nosocomial infection rates was identified during the studies.

For the implementation of the project, the coordination of all those involved was necessary, especially with the “Animals and Society” Chair of the Rey Juan Carlos University and the “PsicoAnimal” Association. The involvement of the hospital management, as well as all the members of the PICU, was essential for the development of the program.

It is important to note that these findings should be taken with caution, considering that our project has several limitations. The lack of a control group makes it difficult to determine if the decrease in pain, fear, or anxiety levels is due to AAT or whether it would in fact occur in conjunction with any other type of non-pharmacological therapy. Subgroup analysis and analysis with exclusion of patients receiving continuous analgesia attempt to control for these confounding variables; however, there may be many others that have not been considered.

Additionally, at the onset, the participants did not have any condition that may have justified any degree of pain, and consequently, the scores on these scales are low, which can make it difficult to draw conclusions about it.

We must also consider that the m-YPAS scale was developed to measure the level of anxiety before entering the operating theater, and not in the PICU environment. Finally, the presence of participants in units other than the PICU means that the sample becomes much more heterogeneous.

## Conclusion

The implementation of an animal-assisted therapy project in a pediatric intensive care unit is feasible and safe and has a high degree of acceptance by participants, caregivers, and healthcare personnel. Although previous studies have not reported a decrease in fear or anxiety scales, this is the first study conducted in a PICU setting that demonstrates a reduction in pain, fear, and anxiety.

The satisfaction shown with this type of therapy is very high and the participants’ recommendation to extend it to other patients supports its inclusion in the near future as part of the approach for humanization and non-pharmacological therapy currently in place in our PICUs.

For the success of this type of therapy, teamwork is essential, with staff involved in all humanizing activities and experts in AAI therapy who guide the sessions and are aware of the animals’ needs. Coordination between the medical team and AAI professionals is mandatory when choosing patients and setting goals; barriers to implementation in terms of concern for increased infections have been ruled out both in this study and in previous literature, and therefore should not continue to be a concern.
